# Assessing parents’ knowledge and attitudes towards seasonal influenza vaccination of children before and after a seasonal influenza vaccination effectiveness study in low-income urban and rural Kenya, 2010–2011

**DOI:** 10.1186/1471-2458-13-391

**Published:** 2013-04-25

**Authors:** Prisca Adhiambo Oria, Geoffrey Arunga, Emmaculate Lebo, Joshua M Wong, Gideon Emukule, Philip Muthoka, Nancy Otieno, David Mutonga, Robert F Breiman, Mark A Katz

**Affiliations:** 1 Kenya Medical Research Institute/Centers for Disease Control and Prevention (KEMRI/CDC), Nairobi, Kenya; 2 Centers for Disease Control and Prevention, Atlanta, GA, USA; 3 Ministry of Public Health and Sanitation, Nairobi, Kenya

**Keywords:** Seasonal influenza, Vaccination, Attitude, Parent, Children, Low-income, Kenya

## Abstract

**Background:**

Influenza vaccine is rarely used in Kenya, and little is known about attitudes towards the vaccine. From June-September 2010, free seasonal influenza vaccine was offered to children between 6 months and 10 years old in two Population-Based Infectious Disease Surveillance (PBIDS) sites. This survey assessed attitudes about influenza, uptake of the vaccine and experiences with childhood influenza vaccination.

**Methods:**

We administered a questionnaire and held focus group discussions with parents of children of enrollment age in the two sites before and after first year of the vaccine campaign. For pre-vaccination focus group discussions, we randomly selected mothers and fathers who had an eligible child from the PBIDS database to participate. For the post-vaccination focus group discussions we stratified parents whose children were eligible for vaccination into fully vaccinated, partially vaccinated and non-vaccinated groups.

**Results:**

Overall, 5284 and 5755 people completed pre and post-vaccination questionnaires, respectively, in Kibera and Lwak. From pre-vaccination questionnaire results, among parents who were planning on vaccinating their children, 2219 (77.6%) in Kibera and 1780 (89.6%) in Lwak said the main reason was to protect the children from seasonal influenza. In the pre-vaccination discussions, no parent had heard of the seasonal influenza vaccine. At the end of the vaccine campaign, of 18,652 eligible children, 5,817 (31.2%) were fully vaccinated, 2,073 (11.1%) were partially vaccinated and, 10,762 (57.7%) were not vaccinated. In focus group discussions, parents who declined vaccine were concerned about vaccine safety or believed seasonal influenza illness was not severe enough to warrant vaccination. Parents who declined the vaccine were mainly too busy [251(25%) in Kibera and 95 (10.5%) in Lwak], or their child was away during the vaccination period [199(19.8%) in Kibera; 94(10.4%) in Lwak].

**Conclusion:**

If influenza vaccine were to be introduced more broadly in Kenya, effective health messaging will be needed on vaccine side effects and frequency and potential severity of influenza infection.

## Background

During influenza seasons, infants and young children have increased hospitalization risk [1,2]. Children also play an important role in spreading influenza; school-children are a principal reservoir from which influenza is introduced to households [3-5]. The main intervention to prevent influenza is vaccination [6-8]. It is important for health educators to understand factors that shape parental views about vaccination because parental attitudes about vaccination are highly associated with rates of childhood vaccination [9,10]. If parents do not view a specific vaccine as necessary or acceptable, vaccination campaigns can be ineffective [11,12].

In Kenya, influenza occurs year-round, with peaks in colder months [13]. Currently, in Kenya, a country of 39 million people, less than 30,000 doses of influenza vaccine are distributed per year, exclusively in the private sector [14]. Public attitudes about influenza and influenza vaccine are largely unknown. Influenza vaccine is not part of routine childhood immunization. Public understanding of the disease burden of influenza, and public appreciation that seasonal influenza vaccine is safe, well tolerated, and effective in healthy children would be important in establishing support for policy decisions concerning routine influenza vaccination [15].

In 2010, Kenya Medical Research Institute/Centers for Disease Control and Prevention (KEMRI/CDC) launched a three-year seasonal influenza vaccine effectiveness study in two pre-existing population-based infectious disease surveillance (PBIDS) sites --Kibera, an informal settlement in Nairobi, and Lwak, a rural community in Nyanza province. Free seasonal influenza vaccine was offered to children, 6 months to 10 years old, enrolled as study participants in the two sites.

To examine parents’ opinions about childhood seasonal influenza vaccination, we carried out focus group discussions and administered a questionnaire to parents before and after the first year of the vaccine campaign.

## Methods

We conducted a quantitative cross-sectional survey by administering standardized questionnaires through individual interviews, and we conducted a qualitative analysis using a series of focus group discussions. The questionnaires and focus group discussion guides were pretested in the study communities before being used for actual data collection.

### Study sites and population

Since 2005, KEMRI/CDC has conducted PBIDS in two low-income sites in Kenya, one within Kibera and the other in Lwak. The surveillance sites together comprise a population of approximately 55,000 people. Kibera is an informal settlement in Nairobi, one of the largest urban slums in Africa. KEMRI/CDC PBIDS is conducted among residents of two of the 13 villages in Kibera, Soweto and Gatwikira. Most employed residents are casual laborers, servants, security guards or small-scale traders. Lwak is a rural location in western Kenya along Lake Victoria. The population is predominantly subsistence farmers and fishermen. As part of PBIDS, community interviewers visit enrolled households every week and ask participants standardized questions about recent illnesses. The PBIDS protocol has been described previously [15].

In 2010, KEMRI/CDC launched a three-year seasonal influenza vaccine effectiveness study among families enrolled in PBIDS in Kibera and Lwak. The study was targeted at young children (6 months of age through 10 years old) based on evidence that influenza was a significant cause of respiratory illness in Kibera and Lwak [14]. Sanofi Pasteur donated the vaccine. Parents brought their child to one of three vaccine administration centers in each PBIDS site during a three-month period (June-September, 2010). Children < 9 years old were offered two doses of the vaccine. The vaccine campaign was preceded by vaccine awareness campaigns using posters, information leaflets, meetings and home visits. Study staff held meetings with community leaders to inform them about and obtain their acceptance of the planned vaccine campaign. In addition, community interviewers visited each household with an eligible child, as determined from the PBIDS database, at least twice and guided caretakers of children through the vaccine information leaflet. Posters were strategically placed in public places such as shopping areas, hospitals and main walking paths.

### Questionnaire

We administered pre- and post-vaccination questionnaires from June 7–13, 2010, and September 27 to October 3, 2010, respectively.

The standardized questionnaire had closed-ended questions; answers were recorded into a pre-programmed (Visual Studio.NET 2005) Personal Digital Assistant (PDA). We asked about reasons for receiving or declining the seasonal influenza vaccine, and perceptions about and sources of information about the vaccine campaign.

We administered a questionnaire one week before and after the vaccine campaign. For the survey, we approached all households enrolled in the two PBIDS sites. We asked the heads of households or their proxy whether they were health decision makers in that household and whether an eligible child lived in the household. We only administered the questionnaire to respondents who answered yes to both questions.

### Focus group discussions

In February 2010, we conducted four pre-vaccination focus group discussions, two each in Kibera and Lwak, with a total of 36 parents. In each site, discussions were separately held for fathers and mothers. During March - April 2011, we conducted 12 post-vaccination focus group discussions, six per site, with a total of 75 parents. In each site, three focus group discussions were with fathers and three with mothers.

Focus group discussion sessions lasted between 45 and 90 minutes and were facilitated by behavioral scientists from KEMRI/CDC. During the sessions, one investigator moderated while another collected field notes. Before each focus group discussion started, the moderator provided a brief description of the study and obtained written consent and permission to audio record the session from the participants.

Facilitators followed a discussion guide for each session. The pre-vaccination focus group discussion guide included questions about knowledge, attitudes and practices related to seasonal influenza virus infection and vaccination of children, and sources of health-related information. The post-vaccination focus group discussion guide included questions on attitudes towards seasonal influenza virus infection, motivators and barriers to vaccination, experiences with past vaccination campaigns, and sources of vaccine campaign information. Investigators constructed questions using expert opinion, literature reviews and prior research experience with the study communities.

For pre-vaccination focus group discussions, we randomly selected mothers and fathers who had an eligible child from the PBIDS database to participate. For the post-vaccination focus group discussions, we stratified parents whose children were eligible for vaccination as fully vaccinated, partially vaccinated, and non-vaccinated. We recruited parents to participate from each of these three strata. Fully vaccinated children were 6 months – 8 years old and received 2 doses or were 9–10 years old and received one dose. Children 6 months – 8 years old were partially vaccinated if they received only 1 dose. Parents who had children with different vaccination statuses (fully, partially and non-vaccinated) were asked to exclusively talk about their child who fell under the specific group for which they were selected. For the questionnaire respondents, we ascertained vaccination status through self-reporting. For the focus group discussions, vaccination status was ascertained through abstraction from the study database from which respondents were assigned to the various vaccination status groups.

### Data analysis

Statistical analysis was performed using Stata 9.2 (StataCorp, College Station, Texas, USA). Recordings from the focus group discussions were transcribed by the study team. A qualitative content analysis identified key themes using verbatim transcripts. A coding framework was used to code the transcript and guide thematic analysis. Throughout the analysis process, emergent themes were added to the coding framework to ensure completeness.

### Ethical review

The seasonal influenza vaccine effectiveness study protocol, of which the questionnaire surveys and the post-vaccination group discussions were a component, was approved by the KEMRI Ethical Review Committee (ERC) (SSC Protocol # 1780). The CDC Institutional Review Board (IRB) formally deferred to the KEMRI ERC for this review (CDC IRB Protocol # 5933). The pre-vaccination discussions were part of a protocol exempted by Kenyan Ministry of Public Health and Sanitation and CDC IRB because of its relevance to the public health response to the 2009 H1N1 influenza pandemic. We obtained verbal informed consent from both groups of participants. Written informed consent had already been obtained from all respondents to participate in PBIDS, the platform on which the vaccine campaign was based.

## Results and discussion

### Quantitative results

#### Pre-vaccination results

We approached 5,822 residents, including 3,731 in Kibera and 2091 in Lwak. Among them, 3,193(86%) in Kibera and 2091(100%) in Lwak had a child aged less than 10 years in the household and made healthcare decisions for children in their household. Only these respondents were interviewed.

Overall, 89.5% respondents in Kibera and 95% in Lwak said they intended to vaccinate their children. Respondents who intended to have their children vaccinated said they intended to do this because seasonal influenza vaccine would protect their children from seasonal influenza (77.6% in Kibera; 89.6% in Lwak), and vaccination of the child would protect the child’s family from seasonal influenza (6.9% in Kibera; 18.4% in Lwak). Also, 11.7% of Lwak respondents and 2.6% of Kibera respondents said the vaccine being free was a reason their child would get the vaccine. Those who said they would not have their children vaccinated (5.6% in Kibera and 3.1% in Lwak), cited the following reasons most commonly: need for more information about the vaccine (10% in Kibera; 73.8% in Lwak), and concerns about the side effects of the vaccine (5% in Kibera; 12.3% in Lwak) (Table 1).

**Table 1:** Responses to pre-vaccination campaign questionnaire about influenza and influenza vaccine, Kibera and Lwak, 2010

**Description**	**LWAK**	**%**	**KIBERA**	**%**	**TOTAL**	**%**
	**Number**		**Number**		**Number**	
**Household has children aged 10 years or younger?**						
Yes	2,091	100.0	3,540	94.9	5,631	96.7
No	0	0.0	191	5.1	191	3.3
**N**	**2,091**	**100.0**	**3,731**	**100.0**	**5,822**	**100.0**
**Do you make, or are you involved in making, healthcare decisions for children in this household?**						
Yes	2,091	100.0	3,193	85.6	5,284	90.8
No	0	0	538	14.4	538	9.2
**N**	**2,091**	**100.0**	**3,731**	**100.0**	**5,822**	**100.0**
**Did you know that a free flu vaccine will be offered in your community starting next month?**						
Yes	769	36.8	2,954	92.5	3,723	70.5
No	1,322	63.2	238	7.5	1,560	29.5
Missing	0	0.0	1	0.0	1	0.0
**N**	**2,091**	**100.0**	**3,193**	**100.0**	**5,284**	**100.0**
**Do you plan to have your child/children get this flu vaccine?**						
Yes	1,986	95.0	2,859	89.5	4,845	91.7
No	65	3.1	180	5.6	245	4.6
Don’t Know	40	1.9	154	4.8	194	3.7
**N**	**2,091**	**100.0**	**3,193**	**100.0**	**5,284**	**100.0**
**Why will you have your child get flu vaccine? (Multiple response)**						
To protect them from flu	1,780	89.6	2219	77.6	3,999	82.5
To protect them from swine flu	94	4.7	379	13.3	473	9.8
To protect my family from flu	365	18.4	196	6.9	561	11.6
The vaccine is free	233	11.7	73	2.6	306	6.3
I heard that children should get the vaccine.	228	11.5	505	17.7	733	15.1
**N**	**1,986**		**2,859**		**4,845**	
**Where did you get information on the flu vaccine? (Multiple response)**						
Doctor/Clinical Officer/Nurse	153	67.1	2	0.4	155	21.1
Community health worker	19	8.3	436	86.3	455	62.1
Religious leader	2	0.9	0	0.0	2	0.3
Friend or neighbor	38	16.7	62	12.3	100	13.6
Radio	5	2.2	0	0.0	5	0.7
Posters	1	4.4	48	9.5	58	7.9
Other	38	16.7	14	2.8	52	7.1
**N**	**228**		**505**		**733**	
**Why will you NOT have your child get flu vaccine? (Multiple response)**						
I need more information	48	73.8	18	10.0	66	26.9
Flu is not a serious health concern	2	3.1	2	1.1	4	1.6
I worry about adverse effects of flu vaccine	8	12.3	9	5.0	17	6.9
I worry about adverse effects of vaccines	4	6.2	2	1.1	6	2.4
I don’t think it gives protection	6	9.2	1	0.6	7	2.9
The vaccine may give them flu	1	1.5	0	0.0	1	0.4
There is treatment if my child gets flu	1	1.5	1	0.6	2	0.8
Other	0	0.0	147	81.7	147	60.0
**N**	**65**		**180**		**245**	

#### Post-vaccination results

At the end of the vaccine campaign, of 18,652 eligible children aged 6 months – 10 years, 5,817(31.2%) were fully vaccinated, 2,073(11.1%) were partially vaccinated and, 10,762(57.7%) were not vaccinated. For the post-vaccination survey, we approached 8,532 residents, including 3,598 in Kibera and 4,934 in Lwak. Out of those, 3,185(89%) in Kibera and 2,570(52%) in Lwak, had a child aged less than 10 years in the household and made healthcare decisions for children in their household and were interviewed.

Among the interviewed residents, 64.4% in Kibera and 63.4% in Lwak said that at least one of their children had been vaccinated. Respondents who did not vaccinate their children (31.5% in Kibera; 35.2% in Lwak) cited the following reasons most commonly: parents were too busy (25% in Kibera; 10.5% in Lwak); the child was away during the vaccination period (19.8% in Kibera; 10.4% in Lwak); and inconvenient vaccination hours (10.6% in Kibera; 7.9% in Lwak). Additionally, 3.2% of Lwak respondents and 2.1% of Kibera respondents said that the vaccine being free was a reason for getting their child vaccinated. Also, 2.4% of Lwak respondents and 2.5% of Kibera respondents said their children did not get the vaccine because they did not know the vaccine was free (Table 2).

**Table 2:** Responses to post-vaccination campaign questionnaire about influenza and influenza vaccine, Kibera and Lwak, 2010

**Description**	**LWAK**	**%**	**KIBERA**	**%**	**TOTAL**	**%**
	**Number**		**Number**		**Number**	
**Household has children aged 10 years or younger?**						
Yes	3,036	61.5	3,514	97.7	6,550	76.8
No	1898	38.5	82	2.3	1,980	23.2
Missing	0	0.0	2	0.1	2	0.0
**N**	**4,934**	**100.0**	**3,598**	**100.0**	**8,532**	**100.0**
**Do you make, or are you involved in making, healthcare decisions for children in this household?**						
Yes	2,570	84.7	3,185	88.5	5,755	86.8
No	466	15.3	329	9.1	795	12.0
Missing	0	0.0	84	2.3	84	1.3
**N**	**3,036**	**100.0**	**3,598**	**100.0**	**6,634**	**100.0**
**Did you know that a free flu vaccine was offered to children in your community over the past 3 months?**						
Yes	2,478	96.4	2,836	89.0	5,314	92.3
No	92	3.6	342	10.7	434	7.5
Missing	0	0.0	7	0.2	7	0.1
**N**	**2,570**	**100.0**	**3,185**	**100.0**	**5,755**	**100.0**
**Did any of your children get flu vaccine?**						
Yes	1630	63.4	2,050	64.4	3,680	63.9
No	905	35.2	1,004	31.5	1,909	33.2
Don’t Know	35	1.4	131	4.1	166	2.9
**N**	**2,570**	**100.0**	**3,185**	**100.0**	**5,755**	**100.0**
**Why did you have your child get flu vaccine? (Multiple response)**						
To protect them from flu	1,369	84.0	1,645	80.2	3,014	81.9
To protect them from swine flu	95	5.8	220	10.7	315	8.6
To protect my family from flu	116	7.1	68	3.3	184	5.0
The vaccine was free	52	3.2	43	2.1	95	2.6
I heard that children should get the vaccine.	367	22.5	237	11.6	604	16.4
Because the vaccine offers lifetime protection	131	8.0	00	0.0	131	3.6
**N**	**1,630**		**2,050**		**3,680**	
**Where did you get information on the flu vaccine? (Multiple response)**						
Doctor/Clinical Officer/Nurse	27	7.4	13	5.5	40	6.6
Community health worker	332	90.5	213	89.9	545	90.2
Religious leader	7	1.9	4	1.7	11	1.8
Friend or neighbor	30	8.2	22	9.3	52	8.6
Posters or public notice	30	8.2	20	8.4	50	8.3
Radio	10	2.7	1	0.4	11	1.8
Newspapers	0	0.0	1	0.4	1	0.2
Other	96	26.2	6	2.5	102	16.9
**N**	**367**		**237**		**604**	
**Why did you NOT have your child get flu vaccine? (Multiple response)**						
I didn’t know the vaccine was free	22	2.4	25	2.5	47	2.5
I didn’t know where to get the vaccine	12	1.3	8	0.8	20	1.0
The vaccination hours were not convenient	71	7.8	106	10.6	177	9.3
The queues were too long	44	4.9	23	2.3	67	3.5
I didn’t want my children to miss school	41	4.5	92	9.2	133	7.0
I need more information	57	6.3	0	0.0	57	3.0
Flu is not a serious health concern	10	1.1	0	0.0	10	0.5
I worry about adverse effects from flu vaccine	47	5.2	0	0.0	47	2.5
I worry about adverse effects of vaccines in general	38	4.2	0	0.0	38	2.0
I don’t think it gives protection	6	0.7	0	0.0	6	0.3
I think the vaccine may give them flu	1	0.1	0	0.0	1	0.1
Child not eligible for study	96	10.6	126	12.5	222	11.6
Child had allergy to chicken products	23	2.5	61	6.1	84	4.4
Child was sick during vaccination period	159	17.6	44	4.4	203	10.6
Parent unaware about flu vaccination	52	5.7	90	9.0	142	7.4
Child was away during vaccination period	94	10.4	199	19.8	293	15.3
Vaccination points distant	24	2.7	0	0.0	24	1.3
Parent was too busy	95	10.5	251	25.0	346	18.1
Other	129	14.3	15	1.5	144	7.5
**N**	**905**		**1,004**		**1909**	

### Qualitative results

#### Pre-vaccination results

##### Knowledge and perceptions

Of the 36 pre-vaccination focus group discussion participants, a majority said the main causes of influenza were low temperatures and dust. A few also said smoke, contact with influenza-infected persons and allergic reactions could cause influenza. One father in Kibera explained,

I used to work at a construction site and I would visit the clinic every two weeks with flu. The doctor advised me to stop working at the construction site and my health has since improved. I think it is dust and dampness that causes flu.

Some respondents said it was unnecessary to seek hospital care for influenza; instead, they used home remedies like drinking hot water or hot lemon solution. Other home remedies mentioned were drinking traditional liquor, drinking ginger and garlic solution, sponging the nose with warm water, sniffing herbal medicine powder, and steaming one’s face with herbal medicines solutions. A few respondents mentioned painkillers and antihistamine medications as influenza remedies. Parents in Kibera and Lwak explained,

Flu attacks differently. Some are serious but others only cause a child to cough. So you have to check what kind of flu it is. If the child has a problem breathing then you should worry and take the child to hospital. But if the child has a runny nose but can still play then there is no need to worry.

I have never heard that a child was admitted in hospital because of flu. They just blow their noses and play about in the home.

While none of the pre-vaccination discussion respondents had heard about seasonal influenza vaccine prior to our survey, some were aware of the existence of 2009 pandemic H1N1 influenza and avian influenza vaccines. All respondents were aware of polio, whooping cough, tetanus and measles vaccines, and said their children had previously received them.

##### Vaccine acceptability

A majority of pre-vaccination respondents were willing to have their children vaccinated. However, almost all respondents said they needed more information before they would vaccinate their children. Respondents required information on safety, efficacy and benefits of the vaccine. A mother in Kibera explained,

Whether or not I accept to vaccinate my child will depend on the information I receive from those promoting the vaccine. I must be told how safe the vaccine is, how the vaccine will benefit my child, and from where the vaccine has come.

Most group discussion respondents who were planning to vaccinate their children wanted to prevent their children from getting influenza. Those who would not vaccinate their children were concerned about vaccine side effects and did not perceive seasonal influenza as severe enough to warrant vaccination.

#### Post-vaccination results

##### Attitudes towards influenza infection and vaccine

While most parents in the fully vaccinated group had no concerns about the vaccine, half the parents in the partially and non-vaccinated groups had concerns about the vaccine; most said they were concerned about side effects because it was a new vaccine. Few parents of partially vaccinated children said they were concerned about side effects because they had heard of a child who had reacted negatively to vaccination. Parents of non-vaccinated children in Kibera and Lwak explained,

I had serious doubts about it. I thought to myself … at the clinic children are given all the vaccines … so what are these others that are being introduced later on. Maybe they have negative effects on children. So, I only took my older child so that I see how he reacts to the vaccine before I considered taking my younger child.

There is always a fear with what you have never seen or used. You want to know who has experienced it.

Some respondents in the non-vaccinated group said they did not vaccinate their children because they did not consider influenza a serious illness and said it was normal to occasionally suffer from influenza. However, most parents whose children were either partially or non-vaccinated said they or their child was away during the vaccination period.

##### Experiences with vaccination campaign

Although all respondents said their children had received all of their required childhood vaccines, many said they heard negative remarks specifically about influenza vaccine in the community after they vaccinated their children. Many were told by other parents that they were too quick to accept new things, that their children were being used to test the vaccine, and that the vaccine would make their child sick or the vaccine would kill their child. A mother of a fully vaccinated child in Lwak explained,

There are people who did not welcome this vaccination campaign. If they heard that you were taking your child to be vaccinated they would say you are the people taking your children to be used to test the drug that had been brought by the white man.

##### Impact of cost

Few respondents knew the cost of seasonal influenza vaccine. After being informed of the price (US $11/1000 Kshs), some parents of fully and partially vaccinated children said they would pay for the vaccine in the future if it was offered for a fee. All parents in the non-vaccinated group said they would never pay for the vaccine because they could not afford it and influenza was not a serious illness.

## Discussion

This is one of the first studies to evaluate attitudes about influenza infection and vaccine in low-income urban and rural communities in sub-Saharan Africa. The study contains useful information for those considering future vaccination efforts. The relatively high acceptance of free influenza vaccine in these two communities is encouraging, and suggests that acceptance would not be a barrier to introducing seasonal influenza vaccine on a broader scale in Kenya. One reason for the high acceptance may relate to the appreciation and acceptance of vaccines in general in Kenya and the fact that the vaccine was offered free of charge. Many respondents said their children had received many non- influenza routine vaccines in the past.

Before the vaccination campaign, respondents who were not planning to get their child vaccinated mainly cited concerns about vaccine safety and efficacy and interest in receiving more information. Group discussion participants raised concerns about vaccine safety and efficacy. Other studies assessing parental attitudes towards childhood vaccination have demonstrated that other parents have raised similar concerns [16-18]. A survey of healthcare workers in Kenya prior to a pandemic H1N1 vaccine campaign found concerns about vaccine side effects were a barrier to vaccination [19]. In the remaining two years of our vaccine effectiveness campaign, and in future vaccine campaigns in vaccine-naive communities, strong efforts should be made to educate parents about the very low side effect profile of trivalent seasonal influenza vaccine [20,21].

In interviews conducted after the vaccination campaign, many parents of non-vaccinated children said they did not get their child vaccinated because they were too busy to take their child to the vaccination centers, their child was away or the vaccination hours were inconvenient. We did not use any schools as vaccination centers because all schools in the two PBIDS sites included non-PBIDS residents, and the vaccine supply for this project was limited. Future influenza vaccine campaigns should consider using primary schools as vaccination sites.

Respondents expressed a wide range of opinions about the causes of and treatments for influenza virus infection. In the pre-vaccination discussions, participants cited low ambient temperatures, dust, dirt, and smoke among causes of influenza. Although the former has been shown to be associated with influenza transmission globally [22,23], the latter three factors have not. However, the mention of dust and dirt could reflect an understanding that poor hygiene and indoor air pollution (i.e. from cooking) could lead to increased disease transmission. In addition, respondents mentioned traditional liquor, hot water, hot lemon solution, and ginger and garlic solutions as remedies for influenza. Oseltamivir, which costs at least US $30 for a treatment course at pharmacies in Kenya, would rarely be affordable for Kibera and Lwak residents.

Some residents said they would not vaccinate their children because they doubted the potential severity of influenza and therefore the need for a vaccine. Influenza has been shown to cause severe disease and death in children in the US and other countries [24-27] and pandemic H1N1 influenza caused hospitalizations and deaths in Kenya and other countries in sub-Saharan Africa [24]. Results from influenza surveillance systems in Kenya and other Sub-Saharan African countries are beginning to shed light on the role of influenza in morbidity and mortality in Sub-Saharan Africa [14]; communicating the data, when it is available, to the general public to put influenza burden in proper perspective, may impact demand for vaccination.

Our surveys had limitations. They were conducted in two relatively small communities, so the findings may not be generalizable to the whole country. However, we included low-income rural and urban communities in large population areas, and these populations are likely similar to much of the country. Second, participants were people enrolled in the KEMRI/CDC PBIDS, and their attitudes towards the overall surveillance system may have affected their attitudes towards the vaccine. Third, we did not provide an option to write responses under the “other” category to the question, “Why will you not have your child get the flu vaccine?” in the pre-vaccination questionnaire, and yet we ended up with many respondents choosing the “other” response. Fourth, the number of persons eligible to be interviewed significantly dropped during post-survey especially in Lwak. We suspect that because Lwak is a rural agricultural area, more primary caretakers were away from the homesteads conducting farming-related activities during the post-vaccine interview period. Finally, because of the design of our survey we could not evaluate the effectiveness of the awareness campaign that was conducted before the vaccine campaign. Despite these limitations, our findings offer insights into how parents made decisions to vaccinate their children in two diverse communities in Kenya.

## Conclusion

In both the urban and rural sites, many residents who had not previously heard about seasonal influenza vaccine were willing to have their children vaccinated with a free influenza vaccine. However, these same parents expressed concern regarding potential side effects of the vaccine, and some parents had doubts about the need for vaccine against what they perceived as a mild disease. If influenza vaccine were to be introduced more broadly in Kenya, efforts should be made to inform people about vaccine side effects and the potential severity of influenza infection.

## Competing interests

The authors declare that they have no competing interests.

## Authors’ contributions

PAO participated in the design of the study, made contributions to data collection, interpretation of data, and writing and editing the manuscript. GA and GE participated in the design of the study, performed the statistical analysis, and made contributions to interpretation of data and editing the manuscript. RFB, EL, PM and DM conceived the study and participated in it’s design, and made contributions to interpretation of data and editing the manuscript. NO and JW contributed to data collection, interpretation of results, and editing the manuscript. MAK conceived the study and participated in its design, and contributed to the interpretation of results and to writing and editing the manuscript. All authors read and approved the final manuscript.

## Pre-publication history

The pre-publication history for this paper can be accessed here:

http://www.biomedcentral.com/1471-2458/13/391/prepub
